# Quantitative cardiac MR assessment of left ventricular diastology

**DOI:** 10.1186/1532-429X-15-S1-P27

**Published:** 2013-01-30

**Authors:** Kyle Lehenbauer, Kevin Kalisz, Benjamin H Freed, Xiaoming Bi, Christoph Guetter, Marie-Pierre Jolly, Marius Cordts, Lewis C Sommerville, Keyur Parekh, Michael Markl, James Carr, Jeremy Collins

**Affiliations:** 1 Radiology, Northwestern University, Chicago, IL, USA; 2 Cardiology, Northwestern University, Chicago, IL, USA; 3 Cardiovascular MR R&D, Siemens Healthcare, Chicago, IL, USA; 4 Siemens Corp., Corporate Technology, Princeton, NJ, USA

## Background

Cardiac MR (CMR) is the accepted gold standard for the assessment of myocardial scar and biventricular systolic function. Bright blood cine imaging with phase-contrast mitral inflow assessment has shown promise for the evaluation of left ventricular (LV) diastology. The purpose of this study is to evaluate the accuracy of LV diastolic function grading by CMR, using Doppler echocardiography as the reference standard. We hypothesize that quantitative CMR analysis of diastolic function coupled with left atrial volume (LAV) assessment is accurate in characterizing LV diastology.

## Methods

83 patients without mitral valve disease (47 men, average age 53 years) underwent CMR imaging on a 1.5T scanner for evaluation of myocardial viability or infiltrative heart disease. All patients underwent 2-D phase-contrast (PC) imaging of the mitral valve (TR/TE 48/2.2, FA 30 degrees, VENC 80 cm/s, 20 phases, bw 554). Bright-blood (BB) 4- and 2-chamber cine imaging was performed (TR/TE 13/1.1, FA 69 degrees, 40 to 50 phases, BW 933) for lateral mitral annular and left atrial volume (LAV) assessment. Peak E and A velocities were obtained from PC data. 4-chamber BB cine images were analyzed using prototype software evaluating deformation fields to automatically identify and track the mitral base plane at the lateral and septal insertions, extracting lateral e' values (Siemens Corp., Corporate Technology, Princeton, New Jersey). LAV was obtained using the length-area method. LV diastolic function was graded using established echocardiographic criteria. Differences in means were assessed using the student's t-test.

**Figure 1:**
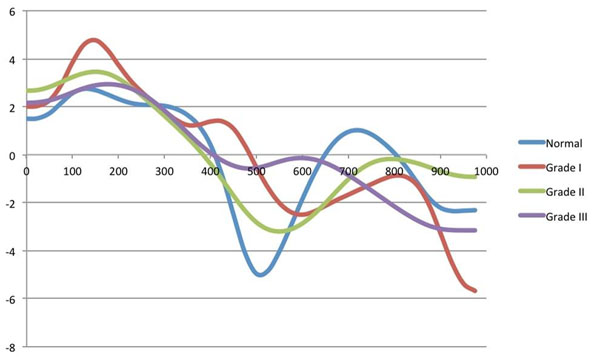
CMR lateral annular e’ velocities in four subjects demonstrating waveforms in patients with normal diastolic function as well as patients with grade I, grade II and grade III diastolic function. Individual patient’s heart rates were extrapolated to 60 bpm for illustration purposes.

## Results

Echocardiographic LV diastolic function was indeterminate in 30 patients (36%). CMR correctly categorized 95%, 80%, 50%, and 50% of patients with normal, grade I, grade II, or grade III LV diastolic dysfunction by echocardiography with an overall accuracy of 77% (Table 1). CMR underestimated echocardiographically determined lateral annular e' and mitral inflow E velocities by 36% and 54% respectively. CMR and echocardiographically determined E:A ratios were not significantly different by diastolic dysfunction grade (p>0.05). E:e' ratios were similar for grade I and III LV diastolic dysfunction (p>0.05), but were statistically different for grade II dysfunction (p = 0.03), with a trend towards higher CMR determined E:e' values (average 16.8 vs 10.5 respectively).

**Table 1:** LV diastolic function CMR classification by echocardiographic diastolic assessment

CMR classification						
Echo classification	Total	Normal	Grade I	Grade II	Grade III	Correctly classified

Normal	22	21	0	1	0	95%
Grade I	15	0	12	3	0	80%
Grade II	12	0	4	6	2	50%
Grade III	4	0	1	1	2	50%

## Conclusions

Quantitative CMR assessment of LV diastolic function is clinically feasible and accurate when using the LAVi to distinguish normal and pseudonormal patterns. Additional work is ongoing to improve CMR grading of LV diastolic dysfunction in the context of an elevated LAVi.

## Funding

Dr. Collins is supported by the RSNA R&E Foundation and the SIR Foundation. Dr. Markl is supported by the NIH on a R01 as well as NUCATS.

